# A comparative study of food habits and body shape perception of university students in Japan and Korea

**DOI:** 10.1186/1475-2891-4-31

**Published:** 2005-10-31

**Authors:** Ruka Sakamaki, Rie Amamoto, Yoshie Mochida, Naotaka Shinfuku, Kenji Toyama

**Affiliations:** 1 Seinan Jo Gakuin University, Faculty of Health and Welfare, Department of Nutritional Sciences, Kitakyusyu, 803-0835, Japan; 2 Seinan Gakuin University, School of Human Sciences, Faculty of Social Welfare, Fukoka, 814-8511, Japan

## Abstract

**Background:**

Abnormal body weight, dietary concerns, and unhealthy weight loss behaviors are increasingly being observed in young females in Japan. Our previous research has shown that the irregular lifestyles of female Japanese and Chinese students are significantly related to their desire to be thinner. In the present study, we compare the food habits and body shape preferences of female university students in South Korea and Japan to explore body shape perceptions in those populations.

**Methods:**

A total of 265 female university students aged 19 – 25 years participated in this study. University students in Korea (n = 141) and university students in Japan (n = 124) completed a self-reported questionnaire. Data were analyzed using SPSS statistical software. Descriptive statistics were used to identify the demographic characteristics of the students and parametric variables were analyzed using the Student's *t*-test. Chi-square analyses were conducted for non-parametric variables.

**Results:**

Comparison of body mass index (BMI) distributions in Japan and Korea showed the highest value in the normal category (74%) together with a very low obesity rate (1.2%). Significant differences were observed between the two countries in terms of eating patterns, with more Japanese eating breakfast daily and with Japanese students eating meals more regularly than Korean students. A difference was also observed in frequency of meals, where Korean students reported eating meals two times per day (59%) and the majority of Japanese students reported eating meals three times per day (81%). Although most subjects belonged to the normal BMI category, their ideal BMI classification was the underweight category (BMI: 18.4 ± 3.4).

**Conclusion:**

Few studies have compared the health related practices of Japanese and Korean university students. The present results suggest the necessity of nutrition and health promotion programs for university students, especially programs emphasizing weight management.

## Background

South Korea has experienced rapid and varied socioeconomic change during the past three decades. Similar to the experience of Japan, the South Korean nutritional transition has also been very rapid. A large increase in the consumption of animal food products and a reduction in total cereal intake have been reported [1]. Also, incidence of metabolic syndrome is now more than 15% in South Korea despite a low prevalence of obesity [2]. Previously, we studied the health related attitudes and body shape perceptions of female Japanese and Chinese university students and compared them with those of other Asian populations [3,4]. Our results showed that despite a very low prevalence of overweight students, the majority of female subjects in both countries have a desire to be thinner.

Nutritional knowledge, food habits, and body-shape preferences vary across cultures. While information about these health-related factors are important for health educators when implementing health-related education programs, little is known about these factors in Korean University students. Therefore, the purpose of this study was to identify and to compare nutritional knowledge, food habits, and body-shape preferences among female university students in Japan and Korea.

## Materials and methods

In 2004, a cross-sectional study was carried out on 150 female Japanese students in Kitakyushu and 130 female Korean students in Seoul. A self-reported questionnaire was administered to 280 students from 19 to 25 years of age. The questionnaire was comprised of three major sections regarding eating, drinking and smoking habits (19 questions), with an additional 4 questions related to body weight. Self-reported height and weight were used to calculate BMI (kg/m^2^). In the present study, we used the BMI classification of the Japan Society for the Study of Obesity (2000) [5] since use of the BMI classification according to the World Health Organization is based on Caucasian populations and is therefore the subject of debate [6,7]. For reference, a comparison of BMI classification according to the Japan Society for the Study of Obesity and the WHO are shown in Table 1. The questionnaire was designed by the authors and is based on a national dietary survey conducted by the Health and Labor Ministry of Japan. Some of the authors also traveled to Korea to investigate the dietary life of Korean people in order to facilitate questionnaire design. The questionnaire was first written in Japanese and then translated to Korean by a native Korean who teaches the Japanese language in Korea. The translated Korean version was then back-translated to insure accuracy. Informed consent was obtained from all participants of this study, according to the Declaration of Helsinki. The statistical software package SPSS 10.0 was used for all data analysis [8]. Parametric variables were analyzed using the Student's *t*-test while chi-squared analyses were conducted for non-parametric variables. All analyses were two-tailed, and 'p' values less than 0.05 were considered statistically significant.

**Table 1:** The comparison of BMI classifications between Japan and WHO. BMI classification of Japan and WHO. Comparison of BMI classification of obesity in Japan and obesity classification according to the WHO

**BMI**	**Japan (2000)**	**WHO (1998)**
<18.5	underweight	underweight
18.5≦-<25	normal weight	normal weight
25≦-<30	obese 1	preobese
30≦-<35	obese 2	obese class I
35≦-<40	obese 3	obese class II
40≦	obese 4	obese class III

## Results

### Sample characteristics and BMI distributions

Survey responses were collected from 95% (265 / 280) of the students. Characteristics of the subjects are shown in Table 2. Of a total of 265 female students, with a mean age of 20 ± 1.9 years, who completed the survey, 124 were Japanese students and 141 were Korean students, with mean ages of 19 ± 1.8 and 20 ± 1.3 years, respectively. The average height was 159.6 ± 5.4 cm, while the average weight was 50.6 ± 6.1 kg. Comparison of BMI distributions between both countries indicated the highest value in the normal category, and a very low rate of obesity (Table 3). According to BMI classifications of the Japan Society for the Study of Obesity (2000), 74.1% of students were classified into the normal BMI range, 24.7% (61/247) students were underweight (BMI < 18.5) and 1.2% (3/247) of students were obese (BMI ≥ 25). No significant difference in BMI was observed between the two countries. The average BMI for Japanese and Korean students was 20.0 ± 2.1 and 19.7 ± 2.0, respectively. Though more Korean students (28.6%) than Japanese students (20.2%) belonged to the underweight BMI category, this observation was not statistically significant.

**Table 2:** Characteristics of participants. Demographic data of study sample. BMI is based on self-reported height and weight. BMI = weight [kg] / height [m]^2^

**Characteristics of Participants**			
	**Total**	**Japan**	**Korea**

**Variable**	n = 248	n = 113	n = 135

**Age (y)**	20.0 ± 1.7	19.0 ± 1.8	20.0 ± 1.3
**weight (kg)**	50.6 ± 6.1	49.6 ± 6.2	51.3 ± 5.9
**height (cm)**	159.6 ± 5.4	157.1 ± 5.2	161.7 ± 4.7
**BMI (kg/m2)**	19.9 ± 2.0	20.0 ± 2.1	20.0 ± 2.1

**Table 3:** BMI distribution of Japanese and Korean university students. The BMI of Japanese and Korean students was categorized into 3 groups (underweight, normal and obese class1), according to the BMI classification of the Japan Society for the Study of Obesity.

**Classification**	**BMI**	**Total**	**Japan (%)**	**Korea (%)**	**p values**
Underweight	<18.5	61(24.7)	23 (20.2)	38 (28.6)	n.s
Normal	18.5≦-<25	183 (74.1)	89 (78.1)	94 (70.7)	n.s
Obese class 1	25≦-<30	3 (1.2)	2 (1.8)	1 (0.8)	n.s

BMI classification as defined by Japan Society for the Study of Obesity (2000)

### Eating habits

Life style practices, in particular food habits, were compared (Table 4). Meal patterns were found to be significantly different between the two countries. Compared to Korean subjects, Japanese reported eating meals more frequently and were also more likely to eat breakfast daily (Japan, 79.0%; Korea, 36.2%; p < 0.01). More than half of the Korean students reported eating meals 2 times per day (58.9%), while the majority of Japanese students (81.0%) eat proper meals three times per day (p < 0.01). Korean students were found to eat fruits and drink alcohol more frequently than Japanese students. Both Japanese (85.4%) and Korean (77.0%) students tend to eat with friends or family members daily or three to four times per week.

**Table 4:** The life style practices of students in Japan and Korea. Table 4 shows the results of questions related to dietary practices with special reference to eating habits. Meal patterns, consumption of fruits and vegetables, consumption of fried foods, consumption of alcohol were assessed for Japan and Korean students. Behavioral differences between two countries were compared using chi-square analyses. Statistical significance was established at p < 0.05.

**Questions**	**Levels**	**Total (%)**	**Japan (%)**	**Korea (%)**	**p values**
Do you take your meals regularly	always regular	120 (45.3)	74 (59.7)	46 (32.6)	**
	irregular	145 (54.7)	50 (40.3)	95 (67.4)	

Do you always take breakfast	daily	149 (56.2)	98 (79.0)	51 (36.2)	**
	three or four times per week	22 (8.3)	3 (2.4)	19 (13.5)	
	once or twice per week	37 (14.0)	12 (9.7)	25 (17.7)	
	rarely	57 (21.5)	11 (8.9)	46 (32.6)	

How many times do you eat meals except snacks	one time	19 (7.3)	5 (4.1)	14 (9.9)	**
	two times	100 (38.2)	17 (14.0)	83 (58.9)	
	three times	141 (53.8)	98 (81.0)	43 (30.5)	
	four times	2 (0.8)	1 (0.8)	1 (0.7)	

How often do you take snacks apart from regular meals	daily	92 (34.7)	41 (33.1)	51 (36.2)	n.s
	three or four times per week	70 (26.4)	28 (22.6)	42 (29.8)	
	once or twice per week	64 (24.2)	34 (27.4)	30 (21.3)	
	rarely	39 (14.7)	21 (16.9)	18 (12.8)	

How often do you eat green, red or yellow colored vegetables	daily	126 (48.1)	70 (56.5)	56 (40.3)	*
	three or four times per week	46 (17.6)	18 (14.5)	28 (20.1)	
	once or twice per week	76 (29.0)	30 (24.2)	46 (33.1)	
	rarely	14 (5.3)	5 (4.0)	9 (6.5)	

How often do you eat fruits	daily	62 (23.6)	18 (14.5)	44 (31.7)	**
	three or four times per week	85 (32.3)	44 (35.5)	41 (29.5)	
	once or twice per week	62 (23.6)	21 (16.9)	41 (29.5)	
	rarely	54 (20.5)	41 (33.1)	13 (9.4)	

How often do you eat fried food	daily	12 (4.6)	9 (7.3)	3 (2.2)	n.s
	three or four times per week	137 (52.1)	67 (54.0)	70 (50.4)	
	once or twice per week	47 (17.9)	29 (23.4)	18 (12.9)	
	rarely	67 (25.5)	19 (15.3)	48 (34.5)	

How often do you take alcohol	daily	2 (0.8)	1 (0.8)	1 (0.7)	**
	two or three times per week	63 (24.2)	18 (14.5)	45 (33.1)	
	rarely	195 (75.0)	105 (84.7)	90 (66.2)	

How often do you eat with friends and family	daily	183 (69.8)	91 (74.0)	92 (66.2)	n.s
	three or four times per week	29 (11.1)	14 (11.4)	15 (10.8)	
	once or twice per week	31 (11.8)	5 (4.1)	26 (18.7)	
	always alone	19 (7.3)	13 (10.6)	6 (4.3)	

Please state your smoking history	current smoker	37 (14.2)	24 19.8	13 9.4	**
	never smoke	223 (85.8)	97 80.2	126 90.6	

What type of food do you think you should eat to have a balanced nutrition	mainly meat	2 (0.8)	1 (1.4)	1 (0.7)	n.s
	mainly vegetable	46 (18.0)	21 (9.1)	25 (18.0)	
	meat, vegetable and other variety of food	181 (70.7)	78 (87.8)	103 (74.1)	
	others	27 (10.5)	17 (1.7)	10 (7.2)	

### Differences between current and ideal body weight

Subjects were asked to report a healthy body weight and ideal body weight for their current height (Table 5). Prior to completing the survey, it was explained to respondents that healthy weight maintains the health and wellbeing of individuals while ideal body weight refers to a desired body image figure. Most respondents were found to have a BMI in the normal BMI category. Students indicated a healthy BMI for their current height as 19.2 ± 1.3 for Japanese students and 18.8 ± 4.2 for Korean students. Healthy BMI of Japanese subjects (19.2 ± 1.3) was almost 1 point higher than the healthy BMI of Korean subjects (18.4 ± 4.4). Students' ideal BMI for their current height was also obtained (Japan: 18.4 ± 1.1, Korea: 18.4 ± 4.4); however, no significant differences were observed between the two countries. On average, the ideal weight was 4.0 kg (Japan) and 3.1 kg (Korea) lower than current weight. It should be noted that respondents' ideal BMI values can be classified into the underweight BMI category.

**Table 5:** Differences between ideal and current body weight

**Variable**	**Total**	**Japan**	**Korea**	**p values**
	n = 248	n = 113	n = 135	
**Current body weight(kg)**	50.6 ± 6.1	49.6 ± 6.2	51.5 ± 5.9	n.s
**Healthy body weight (kg)**	48.5 ± 8.7	47.6 ± 4.4	49.3 ± 11.0	n.s
**Healthy BMI calculated from current height(kg/m^**2**^)**	19.0 ± 3.3	19.2 ± 1.3	18.8 ± 4.2	n.s
**Ideal body weight (kg)**	47.0 ± 8.9	45.6 ± 4.0	48.2 ± 11.3	n.s
**Ideal BMI calculated from current height(kg/m^**2**^)**	18.4 ± 3.4	18.4 ± 1.1	18.4 ± 4.4	n.s

## Discussion

This study aimed to determine and compare the dietary behavior and body shape perception of university students in Japan and Korea. Accordingly, we recorded the distribution of BMI among Japanese and Korean students and found a significantly low prevalence of obesity, a finding that is consistent with a study of Chinese and Japanese students (BMI ≥ 25 overweight 5.8%; BMI > 30 obese 0%) [4]. Previous reports [9,10] have also indicated a low prevalence of obesity in South Korean adults. As South Korea's economic growth accelerated during the past 3 decades, life style changes have included a unique nutrition transition [9]. Although fast food has become very popular among young Koreans, the traditional dietary patterns and intake of staple foods have been maintained at a higher rate than other Asian countries. A report from the Korea National Health and Nutrition Examination Survey (1998) indicated that the rate of overweight (BMI ≦ 25.0 to < 30.0) and obese (BMI ≦ 30) individuals were low among Korean adults; 23.4% and 1.7% in men and 24.9% and 3.2% in women, respectively. However, high rates of diabetes, hypertension, and dyslipidemia were noted in middle-aged and elderly Koreans, even among individuals with relatively low BMI [10]. According to The National Nutrition Survey in Japan (J-NNS), Japan also experienced dietary change from 1950 to 1970 as a result of rapid economic growth. During the past 50 years, the diet of Japanese people has changed remarkably, with the proportion of fat intake in total energy rising to more than 25% [11]. Our results show a low prevalence of overweight and obese conditions among young female subjects in Japan and Korea; however, health issues related to these conditions certainly exist in the middle-aged and elderly generations. Thus, the importance of health promotion at the disease prevention stage can not be overstated, and similar health education programs should be implemented for university students.

The present research shows that meal patterns for the two countries were significantly different. Japanese students reported eating meals regularly and eating breakfast daily. In contrast, Korean students were significantly less likely to eat breakfast daily and ate meals less frequently. South Korea has shown a unique nutrition transition. A range of government and nutrition specialists have made efforts to retain the traditional diet in Korea. This has resulted in a high consumption of vegetables and low level of fat intake [1]. However, few reports have been published to date regarding the food habits and nutrition knowledge of young adults. Publicity and education programs at schools should also emphasize that a healthy eating pattern parallels the beneficial effects of traditional foods.

Our results revealed that Japanese and Korean students desire body weights that are lower than their actual body weight, with Japanese students desiring thinner figures than Korean students. Similarly, previous research on young Japanese women reported that an ideal weight for their current height was an average of 5.2 kg less than current weight [12]. Body shape perception and ideal body shape are strongly influenced by socioeconomic factors. In western society, many young females are extremely concerned with their body weight and shape. Mass media and pictures in fashion magazines have a strong impact on girls' perceptions of their weight and shape [13]. In addition, weight concern is a predictor of the development of eating disorders of at least subsyndromal severity in young females [14]. Therefore, it is vital that educators guide their students to understand that an ideal weight should take into account optimal physiological function. Instilling young women with this knowledge is of particular importance because excessive weight reduction adversely affects their health and reproductive systems.

## Conclusion

The findings of the present study show that BMI distributions of female students in both Japan and Korea have the highest values in the normal BMI category, together with very low obesity rates. In terms of eating patterns, significant differences were observed between the two countries, with more Japanese students reporting eating meals regularly and eating breakfast daily than Korean students. A difference was also observed in meal frequency, where Korean students reported eating meals two times per day and the majority of Japanese students reported eating meals three times per day. Although most subjects belonged to the normal BMI category, their ideal BMI values were classified into the underweight category. Little research has been carried out comparing the dietary habits and body figure perceptions of Japan and Korean students. The present results and previous data from female Chinese university students show a desire for a thinner figure similar to that observed in western society. These findings suggest the need for improved nutrition education for female students, especially education regarding body weight management.

## Authors' contributions

R.S carried out questionnaire design, manuscript drafting and total coordination of the study. R.A contributed to the data entry and its analysis. Y. M was actively involved in the study's implementation and in data collection. S.N and K.T contributed to final approval of the manuscript.
